# The role of heuristics and biases in cancer-related decisions

**DOI:** 10.3332/ecancer.2013.ed26

**Published:** 2013-09-19

**Authors:** G Pravettoni, A Gorini, B Bonanni, U Veronesi

**Affiliations:** 1 European Institute of Oncology, Milan, Italy; 2 University of Milan, Milan, Italy

**Keywords:** heuristics, biases, decision making, cognition, emotion, genetic, prophylactic surgery

Prophylactic mastectomy to reduce the risk of developing breast cancer in the presence of a BRCA mutation has been available for many years. Yet, immediately after Angelina Jolie’s announcement of her decision to undergo a bilateral prophylactic mastectomy, the level of women’s interest in genetic testing and/or preventive surgical intervention soared incredibly as witnessed by the increased number of information requests followed the announcement. FORCE (http://facingourrisk.wordpress.com/), a non-profit organization dedicated to improving the lives of those affected by hereditary breast and ovarian cancer, reported that calls to their hotline quadrupled within 36 hours. A sudden increased interest in genetic information related to breast cancer was also noted by Wikipedia which announced that the page describing the gene, BRCA 1, was viewed nearly 30,000 times the day of Angelina’s announcement. Moreover, many physicians report that cancer genetic clinics have seen an uptake in women seeking BRCA 1/2 genetic testing following Ms. Jolie’s disclosure.

Cognitive science, more accurately than medicine itself, can provide an explanation for this phenomenon. In fact, according to the descriptive theories of decision, Jolie’s story emphasizes how the role played by heuristics in driving human decisions can become stronger than that played by rationality. “My mother fought cancer for almost a decade and died at 56”, Jolie declared, expressing the personal motivation that led her to take this difficult decision. Tversky and Kahneman [1] would have attributed this choice to the availability heuristic, a mental shortcut occurring when people make judgments about the probability of events by how easy it is to think of examples. According to the availability heuristic, the easier it is to recall or imagine the consequences of something, the greater we perceive these consequences to be. The reason why this heuristic can bias our perception of reality is that the frequencies with which events come to mind are not usually accurate reflections of their actual probability in real life. A woman whose mother died from cancer when only 56 will tend to overestimate (no matter what the actual reality is) both the probability of dying from cancer in the general population, as well as the probability that she herself will die from cancer. She will therefore become more prone to take a drastic decision such as preventive bilateral mastectomy than someone else who has not had the same experience.

A second point emerging from Jolie story regards the psychological effect that a celebrity’s decision can have on other individuals. Using the representativeness heuristic [1] people tend to relate to her and think they have the same problem (and thus tend to look for the same solution) even though their situation is totally unconnected, as in the case of the 90 percent of women with family histories not linked to increased risk of the BRCA genetic mutation that would not benefit at all from genetic counselling or testing. When people rely on representativeness to make decisions, they are likely to judge wrongly because the fact that something is more representative does not necessarily make it more likely. As a consequence of neglecting the relevant base rates and being unable to accurately predict the likelihood of a certain event (such as the probability of developing breast cancer) people will say: “I want to do the genetic test that Angelina had", or “I want to undergo the same surgery she did”, even though such interventions may not be necessary or even applicable to their actual health condition. In addition to creating confusion and false expectations, there may also be serious financial implications for private individuals, for the health care system and for private health insurance companies that, at least in the USA, are likely to pay for the procedure when there is a strong family history of breast and/or ovarian cancer.

The third heuristic emerging from the Jolie story is the anchoring heuristic [1], a cognitive bias occurring when individuals tend to rely too heavily on the first piece of information (the “anchor”) they receive when making decisions. Once an anchor is set, people make their choice by adjusting away from that anchor, often resulting in insufficient adjustments and consequently in biases toward interpreting other information around it [2]. From the Jolie story people have received two different anchors. The first one is numerical: “My chances of developing breast cancer have dropped from 87% to under 5%. I can tell my children that they don’t need to fear they will lose me to breast cancer” she wrote. Although Jolie herself affirmed that “the risk is different in the case of each woman”, the anchor our mind automatically creates is that this particular surgery reduces an 82% risk. The second anchor is more psychological and relates to the first information we received about the current health status of the actress: she is as charming as she has always been, and back to her private and working life just a few days after surgery. This information anchors us just on the positive effects of the operation, without letting us place the right amount of attention on its related risks, difficulties, and possibly later outcomes and side effects.

Relying on heuristics and emotional interpretation of information, the Jolie story biases individuals towards thinking that preventive mastectomy, and preventive surgery in general, is the right choice and will lead to the same positive outcomes for everybody regardless of their illness, clinical and family history or genetic profile. The fallacy of this approach is evident from the fact that, perhaps, a mutation in the BRCA genes not only increases the risk of breast cancer, but also that of ovarian cancer, which is notoriously a much more insidious and deadly disease.

According to the normative theories of decision, in order to make a choice in conditions of risk and uncertainty, a fully rational decisionmaker evaluates the severity and likelihood of all the possible outcomes in order to choose the option with the highest expected value. Such a process is supposed to be entirely guided by rationality, without any emotional interference. On the contrary, numerous observations in the field have shown that humans are characterized by a bounded rationality and that their decisions are often based on feelings and emotions which, whether naturally occurring or experimentally induced, exert a strong influence on individuals’ risk perceptions, risk preferences, and decision making [3–6]. Giving informational inputs into decision making, emotions may influence individuals’ choices directly by evoking specific tendencies toward action [7] and appraisal [3–8] or indirectly by interfering with their ability to retain and process information, thereby impacting comprehension.

Medical decisions, especially those that require important risk evaluation such as in the genetics field, are particularly influenced both by heuristics and emotional interferences. In fact, by nature, humans do not easily manage the concept of risk, not only because of their wellknown innumeracy, but also because emotional reactions usually alter the rational analysis of statistical information. Our mind goes over such information creating many different scenarios imbued with emotions that arise from our past knowledge and experiences, such as the vividness with which consequences can be imagined or remembered. These scenarios alter basic probabilities in favour of a subjective perception of risk based on an affective rather than cognitive evaluation of hazards.

In conclusion, we do not want to underestimate the positive effect of Jolie’s announcement in giving moral support to some women from families with hereditary breast cancer and in breaking taboos about genetic illnesses. Neither do we want to deny the importance of understanding the power of preventive surgery in significantly reducing the risk of developing cancer. Nevertheless, we argue that, in order to help patients make the most effective and rational decisions, best practice and physician communication skills should be focused on the personalization of information with the aim of educating patients about the meaning of multiple personal risks and the pros and cons of the various risk-reducing measures (radical versus conservative) according to their specific health status. Moreover, the development of metacognition abilities that raise the awareness of patients (and physicians!) about heuristics and the effects they can have in altering rational decisions is necessary in order to reduce biased decisions and cognitive distortions. Such knowledge is supposed to prevent erroneous expectations or excessive requests of screening and treatment options that are useless or unrelated to the individual’s personal health status.

The authors did not receive any financial support and have no competing interests.
